# Effect of Lyophilised Sweet Potato on the Quality and Nutritional Characteristics of Set Yogurt

**DOI:** 10.1002/fsn3.70111

**Published:** 2025-03-19

**Authors:** Mehmet KIlinç, Gökhan Akarca, Ayşe Janseli Denizkara

**Affiliations:** ^1^ Asist. Prof. Dr. Faculty of Engineering, Food Engineering Department Afyon Kocatepe University Afyonkarahisar Turkey; ^2^ Assoc. Prof. Dr. Faculty of Engineering, Food Engineering Department Afyon Kocatepe University Afyonkarahisar Turkey; ^3^ Ph.D. Candidate Faculty of Engineering, Food Engineering Department Afyon Kocatepe University Afyonkarahisar Turkey

**Keywords:** antioxidant effect, nutritional value, selenium, tartaric acid

## Abstract

This study examined the functional properties of yogurts produced from two distinct lyophilized dried sweet potatoes and analyzed the temporal changes in those properties during storage. Adding purple and orange sweet potatoes had different effects on the changes in the quality, nutritional, microbiological, and functional properties of yogurt. These effects generally increased in parallel with the added amounts. At the end of storage, it was determined that the samples with 2% purple sweet potato added had the highest dry matter (8.45%), water holding capacity (50.90), DPPH radical scavenging effect (112.97%), and total phenolic substance (2.64 mg GAE/g) values. It was also determined that the richest samples in terms of mineral content were yogurts produced with the addition of 2% purple sweet potato, and the highest minerals were potassium (2,765,987.42 ppb), sodium (250,078 ppb), and magnesium (203,691.47 ppb), respectively. Similarly, it was determined that the textural properties were better in yogurt samples produced with a 2% purple sweet potato addition (the highest firmness (229.07 g) consistency (2690.00 g) cohesiveness (−81.70) and viscosity index (−290.54 g)). On the other hand, the highest oxalic (6.021 mg/kg), tartaric (26.068 mg/kg), formic (10,860 mg/kg), malic (196.11 mg/kg), ascorbic (10.75 mg/kg), and lactic acid (2762.83 mg/kg) values on the last day of storage were found in yogurts produced with a 2% orange sweet potato addition.

## Graphical abstract

The study examined yogurts made from lyophilized dried sweet potatoes and their changes during storage. Sweet potato addition increased acidity, dry matter content, water‐holding capacity, syneresis, DPPH radical scavenging activity, and mineral content.

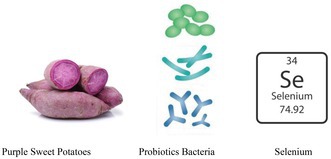

## 1 Introduction

Yogurt is a fermented dairy product that is becoming more and more popular because of its sensory qualities, in addition to being a healthful and nutrient‐dense diet, particularly because of its high protein and mineral content. It is typically made in industrial settings utilizing cow's milk in symbiotic cultures of *Lactobacillus delbrueckii* subsp. *bulgaricus* and *Streptococcus thermophilus* bacteria under carefully regulated temperature and environmental conditions (Tamime and Robinson 1999).

Yogurt not only has a high nutritional content (rich in carbohydrates, protein, fat, vitamins, calcium, and phosphorus) but it is also very digestible and has anti‐disease properties (Ünver 2014). It is particularly recommended for people with gastrointestinal problems including metabolic disorders, lactose intolerance, and inflammatory bowel disease. Furthermore, it enhances immune system performance, lowers the chance of type 2 diabetes, and facilitates weight control (Fisberg and Machado 2015; Sharon & Hutkins 2018; Şanlıer et al. 2019; Cassani et al. 2020).

Prebiotics are healthy food ingredients that the body cannot digest and which, when consumed, encourage the growth of good bacteria in the stomach. One of the most popular food sources of prebiotics is yogurt (Ayar and Gurlin 2014).

One of the earliest known vegetables, sweet potatoes ( *Ipomoea batatas* (L.) Lam.) are high in dietary fiber and provide numerous health benefits for people. Today, they are grown in more than 100 countries with tropical, subtropical, and temperate climates. The tubers are colored in a variety of ways, ranging from cream and white to purple, yellow, and orange (Low et al. 2017). Because of their advantageous protein composition, richness in β‐carotene, antioxidants, anthocyanins, total phenolics, dietary fiber, ascorbic acid, folic acid, vitamins, and minerals, and other nutrients, sweet potatoes are regarded as valuable for human health (Eke‐Ejiofor and Onyeso 2019; Islam 2006; USDA 2007; Tumwegamire et al. 2011).

Research shows that sweet potato tubers exert beneficial effects against many diseases. They have been found to possess antioxidant, antidiabetic, antihyperlipidemic, immunomodulatory, anti‐inflammatory, antitumor, antiobesity, antiaging, hepatoprotective, and antiulcer properties (Ayeleso et al. 2016; Wang et al. 2015; Li et al. 2015; Tsai et al. 2021). Sweet potatoes are also commonly used in traditional medicine to treat high blood pressure, excessive blood sugar, obesity, and cholesterol (Hua et al. 2015).

Sweet potatoes' nutritional advantages and practical qualities have drawn attention to their use in yogurt manufacturing. Anthocyanins, abundant in purple sweet potatoes, are well‐known for their antioxidant qualities and possible health advantages, such as better gut health and weight control. In addition to improving the nutritional profile of sweet potatoes, the fermentation process helps create functional foods like symbiotic yogurt, which blend prebiotics and probiotics to support gut health (Fitriyah 2025).

This study aims to examine changes during storage in the physical, chemical, and microbiological quality characteristics of yogurts made with different additions of lyophilized dried sweet potatoes.

## 2 Materials and Methods

### 2.1 Materials

A dairy company that operates in the marketplaces of Afyonkarahisar, Turkiye, provided raw cow's milk (dry matter: 11.55% (m/v), titratable acidity (lactic acid %): 0.164, pH 6.70, protein: 3.04%, fat: 3.70%) with the right sensory and technological qualities for the creation of yogurt samples. Sweet potatoes (orange and purple) were provided by a manufacturer based in Hatay, Turkiye, and skimmed milk powder was acquired from a private company (Pınar Dairy Products Industry, Izmir, Turkiye) for use in the yogurts' dry matter standardization. The alkaline titration method was used to measure the titratable acidity levels of raw milk samples. The results were expressed as a percentage of lactic acid. A digital pH meter (HI 2215 pH/ORP, Hanna Instruments, Smithfield, RI, USA) calibrated to three different values before use was used to test pH levels.

The gravimetric method was used to determine the dry matter values of raw milk samples used in the yogurt production process. The results are represented as percentages (w/v). The levels of fat are represented as percentages and were measured using a specialized milk butyrometer that rates milk following the Gerber technique (0–8). The protein content was computed by multiplying the nitrogen content (found using the micro‐Kjeldahl method) by 6.38, which was the nitrogen value of the samples that were burned moist. The gravimetric approach was used in a muffle furnace at 550°C to calculate the ash ratio (AOAC 2005).

### 2.2 Starter Culture

A commercial business (Chr. Hansen, Istanbul, Turkiye) provided the Yoflex M780 direct‐vat‐set (DVS) starter culture that contained *Lactobacillus delbrueckii* subsp. *bulgaricus* and *Streptococcus thermophilus* bacteria, which were employed to produce the yogurts for this study.

### 2.3 Lyophilization Process

Using a lyophilizer (LyoQuest, Telstar, Barcelona, Spain) on a lab scale, potato powders were lyophilized. For this process, the potatoes were washed and then peeled and grated with the help of a food processor (AR1044, Arzum, Istanbul, Turkiye). The grated samples were then pre‐frozen at −24°C for 48 h. The sweet potatoes were promptly put in the lyophilizer and freeze‐dried at −45°C at the end of the time. Following a 48‐h lyophilization process, the powdered product was gathered and packaged.

### 2.4 Yogurt Production

The production‐use raw cow milk was heated to 30°C, skimmed milk powder was added, and a refractometer (Atago 2383 Master‐20M, Japan) was used to adjust the dry matter ratio to 12%. Following the process of standardization, the milk was heated to 90°C for 5 min and then allowed to cool to 42°C before the starter culture (M780 starter culture obtained from Chr. Hansen (Hoersholm, Denmark), containing *Streptococcus thermophilus* and *Lactobacillus delbrueckii* subsp. *bulgaricus* strains, bacterial count above 8 log cfu/g) was added. Subsequently, lyophilized sweet potatoes were added at varying rates (0%, 1%, and 2%), thoroughly mixed, and then transferred to sterile 100 mL containers. The containers were subsequently incubated at 43°C ± 2°C in an incubator (INCUCELL, MMM, Planegg, Germany). The yogurts' fermentation was stopped when their pH values reached 4.7. The samples were then chilled for 30 min at room temperature and kept in a refrigerator at 4°C ± 1°C for 10 days or until all analyses were finished.

### 2.5 Analyses

#### 2.5.1 pH and Titratable Acidity

A digital pH meter (HI 2215 pH/ORP, Hanna Instruments) that has previously been calibrated was used to measure the pH values of the yogurt samples (AOAC 2005). The sample titratable acidity levels were determined using the method outlined by Oladipo et al. (2014), expressed as a percentage (m/m) of lactic acid equivalents.

#### 2.5.2 Dry Matter

Using the gravimetric method, the percentage dry matter values of yogurt samples were calculated; the findings were reported as percentages (w/v) (AOAC 2005).

#### 2.5.3 Water Activity (*a*_w_) Values

Yogurt sample water activity (*a*_w_) values were measured using a Lab Touch‐aw instrument (Novasina AG, Lachen, Switzerland).

#### 2.5.4 Analysis of Color Values (L*, a*, b*)

Using a colorimeter (Minolta Co., Osaka, Japan), yogurt sample color values were calculated in accordance with the Hunter color measurement method (Ruiz‐Gutiérrez et al. 2014).

#### 2.5.5 Degree of Syneresis (%)

In order to begin this investigation, 10 g of each yogurt sample was weighed, put on filter paper in a different funnel, and allowed to settle. Following a 10‐min vacuum filtering period for the samples, the amount of yogurt that remained on the filter was weighed, and the percentage of syneresis was determined using the following formula (Wijesinghe et al. 2018):

$$\text{Free Whey}\;\left(\%\right)=\frac{\text{Initial sample weight}-\text{weight of sample after filtration}}{\text{Initial sample weight}}\times 100$$

#### 2.5.6 Water‐Holding Capacity (WHC)

After 12 h of manufacture, the yogurt samples' water‐holding capacity was ascertained. 5 g of yogurt samples were used for this purpose. They were weighed using a precision balance, and they were centrifuged for 30 min at 10°C and 4500 rpm. The pellet that resulted was then weighed after the supernatant was drained off. The following formula was used to determine the samples' percentage WHC (Şengül et al. 2009):

$$\mathrm{WHC}\left(\%\right)=\frac{\text{Pellet weight}\left(g\right)}{\text{Initial weight}\left(g\right)}\times 100$$

#### 2.5.7 Texture Values

Using back extrusion rig hardware, yogurt samples' texture values (firmness, consistency, cohesiveness, and index of viscosity) were assessed using a TA.XT Plus Texture Analyzer (Stable Micro Systems, Godalming, Surrey, UK).

The back extrusion test was performed using a modified method from Buriti et al. (2014). The sample container, measuring 52 mm in diameter and 55 mm in height, was filled with yogurt to a height of 50 mm. The compression disc, measuring 35 mm in diameter, was positioned 50 mm above the sample surface. It was immersed in the yogurt sample to a depth of 30 mm and restored to its initial position. The test was conducted in four replications at a pre‐test speed of 1.0 mm/s, a test speed of 1.0 m/s, and a post‐test speed of 10.0 mm/s.

#### 2.5.8 Determination of Total Antioxidant Compounds

By altering the procedure outlined by Pavithra and Vadivukkarasi (2015), the percentage of DPPH (2‐2‐diphenyl‐2‐picrylhydrazyl) inhibition was used as the basis for the measurement of antioxidant components in potato and yogurt samples. First, 5 g of sample and 25 mL of methanol (LiChrosolv, Merck, Darmstadt, Germany) were added to Falcon tubes, and each tube was vortexed for 2 min (Heidolph Reax Top Vortex, Heidolph Instruments, Schwabach, Germany) and then held at 3°C–8°C for 30 min. The mixture was then centrifuged (Sigma 2‐16 KC, Sigma Laborzentrifugen GmbH, Osterode am Harz, Germany) at 8603 rpm for 30 min. Subsequently, the methanol liquid portion of the samples was passed through special filters (Whatman Grade 40, Sigma‐Aldrich, St. Louis, MO, USA). 100 μL of the sample extract and 100 μL of DPPH solution made in 0.2 mM methanol (Sigma, Germany) were added to a microplate during the analysis. Thermo Scientific Multiskan Sky (Thermo Fisher Scientific, Waltham, MA, USA) was used to detect absorbance at 517 nm after the tubes were left at room temperature for 30 min. Furthermore, three control samples were made, and the absorbance of each was measured. Using the following formula, the results were expressed as a percentage of radical scavenging activity (RSA):

$$\mathrm{RSA}\;\left(\%\right)=\left[\left(\mathrm{Abs}\;\text{control}–\mathrm{Abs}\;\text{sample}\right)/\mathrm{Abs}\;\text{control}\right]\times 100$$

#### 2.5.9 Total Phenolic Contents

To determine the total phenolic content, 25 g of sweet potato powder and yogurt samples were weighed, mixed with 75 mL of 90% ethanol using a magnetic stirrer and then placed in a silent, dark environment for 6 h. The filtrate from these mixtures was then evaporated at 50°C after being filtered through Whatman No. 1 filter paper. After rinsing the flask interiors with distilled water, the mixture was transferred into 25 mL tubes and kept cold until analysis. To measure the total phenolic content, 1 mL of the extracted materials was combined with 46 mL of distilled water and 1 mL of Folin–Ciocalteu reagent using an automatic pipette. Three minutes later, three milliliters of 2% saturated sodium carbonate (Na_2_CO_3_) solution were added, and the mixture was stirred for 2 h in the dark using a magnetic stirrer. At the conclusion of the experiment, the absorbance of the yogurt and sweet potato samples was determined at 760 nm using a spectrophotometer (T60V Spectrometer, PG Instruments, Wibtoft, UK). Using the equation derived from the graph created with the gallic acid standard, the total phenolic contents of a sample were determined as gallic acid equivalent (mg GAE/g sample) (Gülçin et al. 2002).

#### 2.5.10 Organic Acid Analysis

A high‐performance liquid chromatography (HPLC) system (Shimadzu Prominence, Shimadzu Corp., Kyoto, Japan) was used to measure the samples' levels of organic acid. Four grams of yogurt were sampled, and each sample was mixed with 20 mL of 0.01 N H2SO4. Following a vortex, samples were fed into the HPLC system after being passed through 0.45‐μm filters (Guzel Seydim et al. 2000). The system in question had the following characteristics: Activation program: LC Solution; column: Inertsil ODS‐4 (250 mm × 4.6 mm, 5 μm; GL Sciences, Tokyo, Japan); detector: DAD (SPD‐M20A); column oven: CTO‐10ASVp; pump: LC20 AT; autosampler: SIL 20ACHT; mobile phase: ultrapure water, whose pH was brought to 3 with the aid of orthophosphoric acid. The chromatographic separation of these compounds was performed at room temperature. Analysis was run at a flow rate of 1 mL/min with a 10 min run time (Aktaş et al. 2005).

#### 2.5.11 Mineral Substance Analysis

To find the mineral substance levels in the potato and yogurt samples, 0.5 g of dry sample was burned using the wet combustion method (Mars 5, CEM Corporation, Matthews, NC, USA) in a microwave combustion unit with 10 mL of HNO_3_ + H_2_SO_4_. Next, using inductively coupled plasma spectrometry equipment (Vista Series, Varian International AG, Baden, Switzerland), the mineral content in the resulting filtrates was ascertained (Skujins 1998).

#### 2.5.12 Microbiological Analysis

Serial dilutions of yogurt samples were made prior to the microbiological analysis, and the spread plate method was used to analyze the dilutions. Following incubation on *Streptococcus* agar (11007, Merck Millipore, Darmstadt, Germany) and *Lactobacillus bulgaricus* agar (17154, Merck Millipore), respectively, at 42°C for 48–72 h under anaerobic conditions, bacterial counts of *S. thermophilus* and *L. delbrueckii* subsp. *bulgaricus* were carried out (Bracquart 1981).

#### 2.5.13 Statistical Analysis

The study's factorial (5 × 5) design was fully randomized and included replications. The variables were yogurt samples (1% orange sweet potato, 2% orange sweet potato, 1% purple sweet potato, and 2% purple sweet potato) and storage times (1, 4, 7, and 10 days). During the storage period, changes between samples (*p* < 0.05) were found using a two‐way analysis of variance (ANOVA). Correlation analysis was used to evaluate how sample type and storage time interacted. ANOVA and Duncan's multiple range tests were applied to the analysis results using IBM SPSS Statistics 23 (IBM Corp., Armonk, NY, USA).

## 3 Results and Discussion

Table 1 displays the physicochemical characteristics, mineral concentrations, total antioxidant content, and total phenolic content of fresh purple and orange sweet potatoes. Sample type, storage time, and sample type × storage time interactions were highly influential on pH and titratable acidity values (*p* < 0.0001). Furthermore, sample type interactions had a negative effect on pH values and a highly positive effect on titratable acidity values, while storage time interactions had a negative effect on pH values and a highly correlative effect on titration values (Table 2).

**TABLE 1:** Physicochemical properties, mineral content (ppb), scavenging effect of DPPH radical (%), and total phenolic values (mg GAE/g) of fresh purple and orange sweet potatoes.

Analysis	Purple	Orange
pH	6.00	5.93
L* Value	39.13	65.07
a* Value	20.07	26.44
b* Value	−1.63	33.06
Protein (%)	3.38	3.04
Ash (%)	0.1254	0.1106
Dry Matter (%)	4.12	2.48
B	1037.02	1484.36
Na	13,152.3	15,847.17
Mg	501,687.25	310,047.21
K	3,510,004.72	4,164,785.02
Ca	12,102.54	42,163.25
Mn	3314.13	7587.35
Fe	13,458.52	15,884.30
Zn	1587.25	1975.31
Se	1.229	1.089
DPPH	182.24	135.21
TPC	3.07	2.94

Abbreviation: N/D, Not determined.

**TABLE 2:** pH and titratable acidity % values of samples during storage.

Sample	Storage time (d)				Variation		
	1	4	7	10	S	ST	S × ST
*pH*							
Control	4.66 ± 0.01^Aa^	4.54 ± 0.01^Ba^	4.42 ± 0.02^Ca^	4.33 ± 0.02^Da^	*p* < 0.0001	*p* < 0.0001	*p* < 0.0001
1OSP	4.56 ± 0.01^Ab^	4.43 ± 0.01^Bb^	4.37 ± 0.01^Cb^	4.30 ± 0.01^Dab^	*r* = −0.862**	*r* = −0.361*	*r* = n/a
2OSP	4.52 ± 0.01^Ac^	4.37 ± 0.01^Bc^	4.32 ± 0.01^Cc^	4.26 ± 0.01^Db^			
1PSP	4.49 ± 0.01^Ad^	4.43 ± 0.01^Bb^	4.37 ± 0.01^Cb^	4.31 ± 0.03^Dab^			
2PSP	4.47 ± 0.01^Ad^	4.39 ± 0.01^Bc^	4.31 ± 0.02^Cc^	4.28 ± 0.02^Cb^			
*Titratable acidity (% lactic acid equivalents)*							
Control	1.13 ± 0.02^Dd^	1.28 ± 0.01^Cc^	1.40 ± 0.01^Bc^	1.49 ± 0.01^Ab^	*p* < 0.0001	*p* < 0.0001	*p* < 0.0001
1OSP	1.31 ± 0.04^Cc^	1.36 ± 0.01^Cb^	1.45 ± 0.01^Bb^	1.53 ± 0.01^Aa^	*r* = 0.483**	*r* = 0.776**	*r* = n/a
2OSP	1.34 ± 0.02^Cbc^	1.38 ± 0.01^Cb^	1.50 ± 0.01^Ba^	1.56 ± 0.01^Aa^			
1PSP	1.38 ± 0.01^Cab^	1.43 ± 0.01^Ba^	1.42 ± 0.01^Bbc^	1.53 ± 0.01^Aa^			
2PSP	1.42 ± 0.01^Ba^	1.46 ± 0.03^Ba^	1.53 ± 0.01^Aa^	1.54 ± 0.01^Aa^			

*Note:* A–D (→): Values with the different capital letters in the same line for each analysis differ significantly (*p* < 0.05), a—d(↓): Values with the different lowercase letters in the same column for each analysis differ significantly (*p* < 0.05). ± Standard deviation.

Abbreviations: OSP, Orange Sweet Potatoes; PSP, Purple Sweet Potatoes; S, Samples; ST, Storage time.

* Correlation is significant at the 0.05 level (2‐tailed).

** Correlation is significant at the 0.01 level (2‐tailed).

All sample pH values dropped when lyophilized sweet potato was added (*p* < 0.05). The addition of purple sweet potato was more effective on pH values than the addition of orange potato, and the pH value decreased further as the amount added increased. At the beginning of storage, the lowest pH value was 4.47 in the samples to which 2% lyophilized purple sweet potato was added, and the highest pH value was detected in the control samples. The pH value of all yogurt samples showed a decrease during storage (*p* < 0.05). On the last day of storage, the lowest pH value was determined in the samples with the addition of 2% orange potato, with a value of 4.26, and the highest pH value was determined in the control samples with a value of 4.33 (Table 2).

Since the pH value of the sweet potatoes added in the production of yogurts was partially acidic (Table 1), it caused the initial pH values of the samples to decrease and the % titratable acidity values to increase.

Kilinç et al. (2022) reported in their research that lyophilized sweet potato powder added to the frozen mixture reduced the pH value. Similarly, El‐Attar et al. (2022) stated that adding sweet potatoes to yogurt production decreased the pH value more than the control sample. These results are parallel to our findings. The two distinct lyophilized sweet potatoes added to the samples raised their titratable acidity values (*p* < 0.05). While the addition of purple sweet potato had a greater effect on that increase compared to the addition of orange potato, the amount added also had an effect on the increase in titratable acidity. It was observed that the sample with the highest titratable acidity among all yogurt samples on the first day of storage was the sample with the addition of 2% purple sweet potato, with a value of 1.42%, and the lowest was the control sample with a value of 1.13%. Titratable acidity values of all samples increased over the course of the storage period (*p* < 0.05), and on the 10th day of storage, the highest percentage value of titratable acidity in terms of lactic acid was found in the samples supplemented with 2% orange sweet potato (Table 2).

It is stated in the Turkish Food Codex Communiqué on Fermented Dairy Products (Communiqué No. 2009/25) that titratable acidity values in yogurt should be between 0.6% and 1.5% in terms of lactic acid. The results obtained in our study comply with that criterion. Also, Okoye and Animalu (2009) stated that adding sweet potatoes to yogurt increased the titratable acidity value, which is parallel to our research results.

It was found that sample type, storage time, and sample type × storage time interactions were highly effective on the *a*_w_ values of the samples (*p* < 0.0001), whereas storage time and sample type × storage time interactions were highly effective on the % dry matter values (*p* < 0.01). In addition, sample type interactions had a highly negative correlative effect on *a*_w_ values (Table 3).

**TABLE 3:** Water activity (*a*_w_) and dry matter % values of samples during storage.

Sample	Storage time (d)				Variation		
	1	4	7	10	S	ST	S × ST
*a*_w_							
Control	0.806 ± 0.001^Aa^	0.796 ± 0.002^Ba^	0.783 ± 0.001^Ca^	0.758 ± 0.001^Da^	*p* < 0.0001	*p* < 0.0001	*p* < 0.0001
1OSP	0.717 ± 0.001^Ab^	0.702 ± 0.001^Bb^	0.691 ± 0.001^Cb^	0.683 ± 0.001^Db^	*r* = −0.852**	*r* = −0.274	*r* = n/a
2OSP	0.709 ± 0.001^Ac^	0.689 ± 0.001^Bc^	0.680 ± 0.002^Cc^	0.672 ± 0.001^Dc^			
1PSP	0.683 ± 0.001^Ad^	0.676 ± 0.001^Bd^	0.669 ± 0.001^Cd^	0.658 ± 0.001^Dd^			
2PSP	0.673 ± 0.001^Ae^	0.664 ± 0.001^Be^	0.652 ± 0.001^Ce^	0.643 ± 0.001^De^			
*Dry matter (%)*							
Control	14.61 ± 0.01^Cc^	14.83 ± 0.17^BCc^	15.01 ± 0.01^ABd^	15.10 ± 0.01^Ad^	*p* = 0,658	*p* < 0.01	*p* < 0.01
1OSP	15.46 ± 0.37^Ab^	15.73 ± 0.39^Ab^	15.90 ± 0.16^Ac^	16.06 ± 0.06^Ac^	*r* = 0.214	*r* = −0.002	*r* = n/a
2OSP	17.17 ± 0.09^Ba^	17.94 ± 0.12^Aa^	18.11 ± 0.01^Aa^	18.16 ± 0.01^Aa^			
1PSP	15.68 ± 0.38^Ab^	16.04 ± 0.04^Ab^	16.27 ± 0.11^Ab^	16.52 ± 0.24^Ab^			
2PSP	17.65 ± 0.37^Ba^	17.99 ± 0.18^ABa^	18.26 ± 0.08^Aba^	18.45 ± 0.12^Aa^			

*Note:* A–D (→): Values with the different capital letters iabn the same line for each analysis differ significantly (*p* < 0.05), a–e (↓): Values with the different lowercase letters in the same column for each analysis differ significantly (*p* < 0.05). ±Standard deviation.

Abbreviations: S, Samples, ST, Storage time.

** Correlation is significant at the 0.01 level (2‐tailed).

The samples' *a*_w_ values decreased and their dry matter content increased (*p* < 0.05) when lyophilized sweet potato was added. When compared to orange potatoes, purple potatoes were found to be more successful in lowering the *a*_w_ values and raising the amount of dry matter. In addition, in all samples, the *a*_w_ values decreased while the dry matter amounts increased over the course of the storage period (*p* < 0.05); it was found that the highest decrease in *a*_w_ values during storage occurred in the samples supplemented with 2% purple sweet potato, and this was followed by samples supplemented with 1% purple sweet potato and 2% orange sweet potato, respectively. Likewise, samples that had 2% purple sweet potato added to them had the biggest increases in dry matter rates from the start of storage to the finish.

Sweet potato components like starch, fiber, and pectin bind the free water in the mixture, which is why the *a*_w_ values of samples that contained potatoes were lower than those of control samples. The *a*_w_ value of orange sweet potatoes dropped even lower because they bind more free water and have higher compositions of cellulose, lignin, pectin, and hemicellulose than purple sweet potatoes.

Storage time interactions had significant (*p* < 0.01) and highly significant (*p* < 0.0001) effects on the syneresis and water‐holding capacity values of the samples, respectively. Moreover, storage time interactions showed a highly negative correlative effect on syneresis and a positive correlative effect on water‐holding capacity (Table 3).

The samples' syneresis values decreased, and their water‐holding capacity values increased (*p* < 0.05) when sweet potatoes were added. While the addition of purple sweet potato was more effective on the changes in these two values compared to orange sweet potato, the rates of change also increased in parallel with the increase in the amount added. It was determined that the lowest syneresis value at the beginning and during storage was in the samples with 2% purple sweet potato added. These samples were followed by the samples with 2% orange sweet potato added (Table 3).

For every sample, water‐holding capacities rose while syneresis values dropped during storage (*p* < 0.05). The samples generated by adding 2% lyophilized purple sweet potato were shown to have the greatest increase in water‐holding capacity during storage and the greatest decrease in syneresis values.

In their research, Omar et al. (2019) stated that adding sweet potato powder increased the water‐holding capacity of yogurt and decreased the sensitivity to syneresis, which is parallel to our results. Akalin et al. (2012) found analogous findings, noting that an increase in total solids in milk led to enhanced density, reduced pore size in the protein matrix of yogurt gel, decreased syneresis, and improved water‐holding capacity of yogurt gel.

In terms of yogurt quality, it is important that the syneresis value be low. Syneresis is affected by factors such as total dry matter amount, protein and fat contents, denaturation of serum proteins, heat treatment, product storage temperature, acidity development, and the activities of starter cultures (Salvador and Fiszman 2004).

Acidity level affects the structure and syneresis of fermented products such as yogurt. While the water‐holding capacity of proteins is insufficient at low acidity levels (i.e., above 4.6 pH), this property decreases at high acidity (i.e., below 4.6 pH). The water‐holding capacity of proteins increases at pH 4.0–4.6, thereby improving viscosity and decreasing syneresis (Atamer et al. 1986).

In addition to their water‐binding properties, the lyophilized sweet potato powders added to the yogurt samples also reduced the pH of the yogurt thanks to compounds present in their structures, causing a decrease in the degree of syneresis and an increase in water‐holding capacity.

Sample type and storage time interactions had significant (*p* < 0.0001) effects on the DPPH radical scavenging capacities and total phenolic contents of the samples. Furthermore, while sample type had a highly significant positive correlative effect on both of those variables, storage time had a significant negative correlative effect on DPPH radical scavenging capacity and a highly significant negative correlative effect on the total phenolic content (Table 4).

**TABLE 4:** Syneresis and water‐holding capacity values of samples during storage.

Sample	Storage time (d)				Variation		
	1	4	7	10	S	ST	S × ST
*Syneresis (%)*							
Control	37.26 ± 1.03^Aa^	34.75 ± 0.58^Ba^	32.78 ± 0.47^Ca^	31.10 ± 0.37^Ca^	*p =* 0.790	*p* < 0.01	*p* = 0.981
1OSP	35.89 ± 0.33^Aa^	32.30 ± 1.75^Ba^	30.05 ± 0.30^Bb^	29.89 ± 0.97^Ba^	*r* = −0.040	*r* = −0.536**	*r* = n/a
2OSP	31.23 ± 1.27^Ab^	28.98 ± 0.47^Bb^	26.62 ± 0.76^BCc^	24.78 ± 0.56^Cb^			
1PSP	31.94 ± 0.23^Ab^	28.65 ± 1.40^Bb^	26.76 ± 0.54^BCc^	25.64 ± 0.55^Cb^			
2PSP	26.86 ± 0.63^Ac^	25.04 ± 0.01^ABc^	23.67 ± 0.76^Bd^	21.49 ± 1.01^Cc^			
*Water holding capacity*							
Control	32.61 ± 0.65^Cd^	33.78 ± 0.43^BCc^	35.78 ± 0.65^ABb^	38.35 ± 1.64^Ac^	*p* = 0.757	*p* < 0.0001	*p =* 0.974
Control	37.18 ± 0.38^Dc^	40.68 ± 0.21^Cb^	44.57 ± 0.78^Ba^	46.04 ± 0.47^Ab^	*r* = 0.032	*r* = 0.757**	*r* = n/a
1OSP	40.03 ± 0.63^Db^	43.87 ± 0.94^Ca^	46.27 ± 0.08^Ba^	49.74 ± 0.65^Aa^			
2OSP	38.25 ± 0.51^Cc^	41.03 ± 0.30^Bb^	44.90 ± 1.71^Aa^	47.13 ± 0.32^Ab^			
1PSP	43.11 ± 0.81^Da^	45.01 ± 0.49^Ca^	46.69 ± 0.53^Ba^	50.90 ± 0.20^Aa^			

*Note:* A–D (→): Values with the different capital letters in the same line for each analysis differ significantly (*p* < 0.05), a–d (↓): Values with the different lowercase letters in the same column for each analysis differ significantly (*p* < 0.05). ± Standard deviation.

Abbreviations: S, Samples, ST, Storage time.

** Correlation is significant at the 0.01 level (2‐tailed).

The addition of lyophilized sweet potato enhanced the yogurt samples' DPPH radical scavenging capabilities and total phenolic contents (*p* < 0.05), and this impact increased in proportion to the amount added (*p* < 0.05). Furthermore, the addition of purple sweet potato had a greater effect on this value than the addition of orange sweet potato (Table 4). The samples to which 2% purple sweet potato was added had the highest total phenolic contents and DPPH radical scavenging capacities at the start of storage, at 150.95% and 3.35 mg GAE/g, respectively, whereas the control samples had the lowest values, at 69.57% and 2.05 mg GAE/g. DPPH radical scavenging capacities and total phenolic contents of all samples decreased during storage (*p* < 0.05). The highest decrease during storage was observed in the control samples, and the lowest decrease was observed in yogurt samples produced with the addition of 2% purple sweet potatoes (Table 4).

The anthocyanins, phenolic acids, carotenoids, and other compounds found in sweet potatoes caused an increase in the DPPH radical scavenging capacities and total phenolic contents. The decreasing pH during storage caused the breakdown of these compounds, which led to their interaction. Storage time had a highly significant effect (*p* < 0.0001) on the consistency, cohesiveness, and viscosity index values of the yogurt samples, while storage time had a highly significant positive correlative effect on the firmness and consistency values and a highly significant negative effect on the cohesiveness and viscosity index values (*p* < 0.01) (Table 5).

**TABLE 5:** DPPH radical scavenging effect and total phenolic values of samples during storage.

Sample	Storage time (d)				Variation		
	1	4	7	10	S	ST	S × ST
*DPPH radical scavenging effect (%)*							
Control	69.57 ± 2.14^Ad^	64.20 ± 1.39^ABe^	59.66 ± 1.89^BCd^	54.04 ± 2.85^Cc^	*p* < 0.0001	*p* < 0.0001	*p* = 0.018
1OSP	115.50 ± 2.61^Ac^	108.32 ± 2.50^ABd^	101.94 ± 3.43^Bc^	88.51 ± 4.95^Cb^	*r* = 0.807**	*r* = −0.392*	*r* = n/a
2OSP	129.59 ± 4.32^Ab^	126.28 ± 2.35^Ab^	111.94 ± 2.41^Bb^	98.95 ± 1.99^Cb^			
1PSP	121.55 ± 2.80^Abc^	117.54 ± 2.71^Ac^	108.84 ± 0.68^Bbc^	91.75 ± 2.12^Cb^			
2PSP	150.95 ± 9.06^Aa^	150.19 ± 2.58^Aa^	130.52 ± 4.89^Ba^	112.97 ± 7.63^Ba^			
*Total phenolic values (mg GAE/g)*							
Control	2.05 ± 0.05^Ad^	2.01 ± 0.01^Ad^	1.99 ± 0.03^Ad^	1.85 ± 0.01^Bd^	*p* < 0.0001	*p* < 0.0001	*p* = 0.002
1OSP	2.65 ± 0.13^Ac^	2.56 ± 0.12^Ac^	2.46 ± 0.06^Ac^	2.10 ± 0.04^Bc^	*r* = 0.798**	*r* = −0.482**	*r* = n/a
2OSP	2.98 ± 0.06^Ab^	2.82 ± 0.08^ABb^	2.68 ± 0.04^Bb^	2.40 ± 0.05^Cb^			
1PSP	3.08 ± 0.04^Ab^	2.90 ± 0.08^Ab^	2.59 ± 0.09^Bbc^	2.23 ± 0.09^Bbc^			
2PSP	3.35 ± 0.04^Aa^	3.21 ± 0.07^ABa^	3.08 ± 0.07^Ba^	2.64 ± 0.13^Ca^			

*Note:* A–D (→): Values with the different capital letters in the same line for each analysis differ significantly (*p* < 0.05), a–e (↓): Values with the different lowercase letters in the same column for each analysis differ significantly (*p* < 0.05). ± Standard deviation.

Abbreviations: S, Samples, ST, Storage time.

* Correlation is significant at the 0.05 level (2‐tailed).

** Correlation is significant at the 0.01 level (2‐tailed).

While the addition of sweet potato increased the firmness (g), consistency (g·s), and viscosity index (g·s) values of the samples, it decreased their cohesiveness (g) values (*p* < 0.05). While the addition of purple sweet potato was more effective on the changes in these values than the addition of orange potato, the changes in values increased in parallel with the increase in the amount added (*p* < 0.05).

While the firmness (g) and consistency (g·s) values of the samples decreased during storage, the cohesiveness and viscosity index (g·s) values increased (*p* < 0.05) (Table 5). On the final day of storage, the highest firmness, consistency, and viscosity index values were found in the samples to which 2% sweet potato was added, and the lowest values were found in the control samples.

Saleh et al. (2020) stated in their studies that adding sweet potato increased the viscosity in yogurts and improved the yogurt texture. These results align with the findings of our research.

The inclusion of lyophilized sweet potato results in increases in firmness, consistency, and viscosity index values due to the combined effects of the aqueous phase of soluble matter and the insoluble fibers' contribution to the rise in total dry matter. Sweet potatoes include cellulose, lignin, pectin, hemicellulose, and fiber, among other compounds, which bind free water in yogurt and lower its serum content, increasing its hardness, consistency, and viscosity index values. These two values increased more in purple sweet potatoes than in orange sweet potatoes because they contain higher amounts of cellulose, lignin, pectin, and hemicellulose.

**FIGURE 1:**
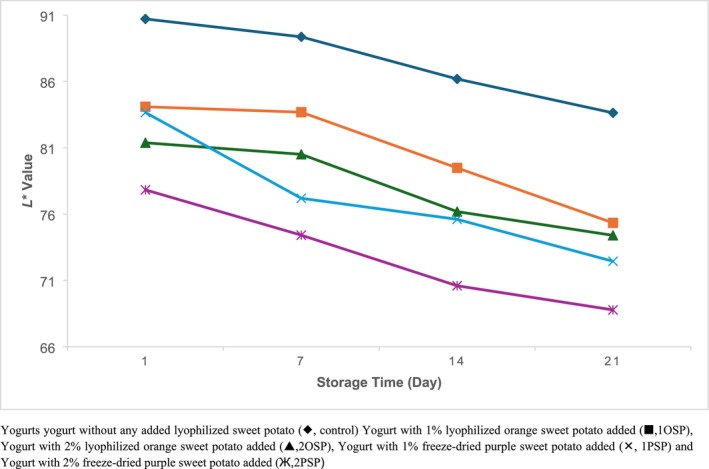
Change L* values of samples during storage. Yogurt without any added lyophilized sweet potato (♦, control), yogurt with 1% lyophilized orange sweet potato added (■, 1OSP), Yogurt with 2% lyophilized orange sweet potato added (▲, 2OSP), Yogurt with 1% freeze‐dried purple sweet potato added (×, 1PSP), and Yogurt with 2% freeze‐dried purple sweet potato added (Ж, 2PSP).

**FIGURE 2:**
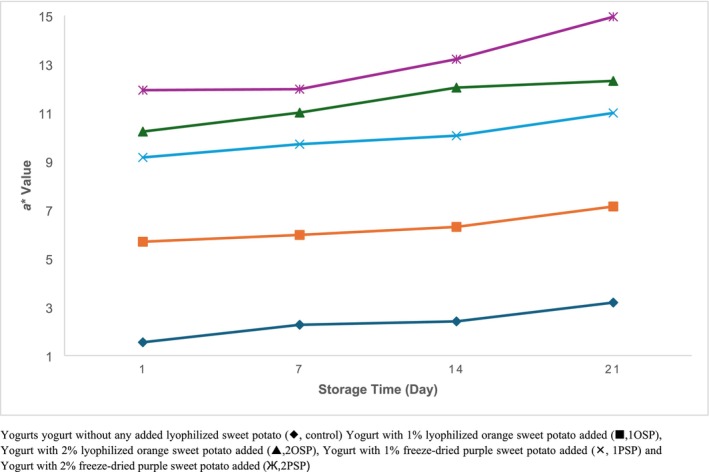
Change a* values of samples during storage. Yogurt without any added lyophilized sweet potato (♦, control), yogurt with 1% lyophilized orange sweet potato added (▀, 1OSP), Yogurt with 2% lyophilized orange sweet potato added (▲, 2OSP), Yogurt with 1% freeze‐dried purple sweet potato added (×, 1PSP), and Yogurt with 2% freeze‐dried purple sweet potato added (Ж, 2PSP).

**FIGURE 3:**
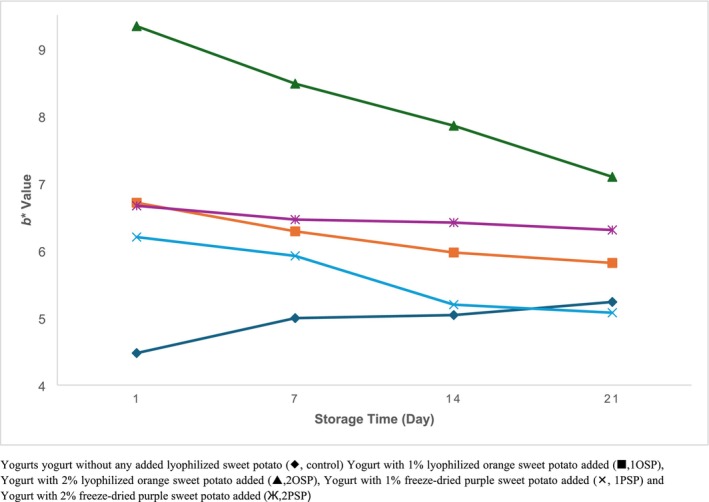
Change b* values of samples during storage. Yogurt without any added lyophilized sweet potato (♦, control) Yogurt with 1% lyophilized orange sweet potato added (■, 1OSP), Yogurt with 2% lyophilized orange sweet potato added (▲, 2OSP), Yogurt with 1% freeze‐dried purple sweet potato added (×, 1PSP), and Yogurt with 2% freeze‐dried purple sweet potato added (Ж, 2PSP).

Figures 1, 2, 3 show that the addition of lyophilized sweet potato to yogurt samples resulted in an increase in a* and b* values (*p* < 0.05) and a decrease in L* values. The samples containing 2% purple sweet potato had the lowest L* value on the first day of storage (77.83), while the control samples had the highest L* value (90.72). Similarly, the samples with 2% purple sweet potato added had the greatest a* value of 11.92 on the first day of storage, whereas the samples with 2% orange sweet potato added had the highest b* value of 9.33.

During the storage period, L* values decreased and a* values increased in all samples (*p* < 0.05). Although b* values increased in the control samples, they decreased in the other samples (*p* < 0.05) (Figures 1, 2, 3).

El‐Attar et al. (2022) stated that the L* value decreased in yogurts with sweet potatoes added, whereas the a* value also decreased.

The changes in the color values of the potato‐containing samples compared to control samples were due to the presence of color substances present in orange and purple sweet potatoes. The decrease in b* values in particular during storage was due to the degradation of anthocyanins, which form purple tones, as a result of decreasing pH values during storage.

Sample type interactions were highly effective on the *S. thermophilus* counts of yogurt samples (*p* < 0.0001), while storage time was effective on *L. delbrueckii* subsp. *bulgaricus* counts (*p* < 0.05) and sample type × storage time interactions were highly effective on the same (*p* < 0.0001). Furthermore, sample type interactions had a highly significant positive correlative effect on *S. thermophilus* counts (Table 6). The addition of sweet potatoes increased the bacterial counts of *S. thermophilus* and *L. delbrueckii* subsp. *bulgaricus* in yogurt samples (*p* < 0.05). While the addition of orange sweet potato was more effective on yogurt bacterial counts compared to purple sweet potato, bacterial counts also increased in parallel with the increase in the amount added (*p* < 0.05; see Table 7).

**TABLE 6:** The textural values of samples during storage.

Sample	Storage time (d)				Variation		
	1	4	7	10	S	ST	S × ST
*Firmness (g)*							
Control	186.15 ± 2.14^Ad^	158.79 ± 6.30^Be^	157.78 ± 2.42^Bc^	128.94 ± 2.60^Cd^	*p =* 0.784	*p* = 0.06	*p* = 0.999
1OSP	211.69 ± 4.30^Ac^	175.33 ± 1.68^Bd^	163.25 ± 1.77^Cc^	159.43 ± 4.11^Cc^	*r* = 0.040	*r* = 0.542**	*r* = n/a
2OSP	290.65 ± 4.37^Aa^	261.44 ± 11.48^Bb^	203.16 ± 4.02^Cb^	189.42 ± 3.89^Cb^			
1PSP	261.44 ± 11.48^Ab^	233.19 ± 3.59^Bc^	192.83 ± 3.47^Cb^	163.94 ± 2.41^Dc^			
2PSP	300.32 ± 5.67^Aa^	290.65 ± 4.57^Ba^	259.24 ± 4.88^Ca^	229.07 ± 5.93^Aa^			
*Consistency (gs)*							
Control	1962.55 ± 83.30^Ac^	1933.86 ± 40.30^Ac^	1707.79 ± 133.74^ABc^	1493.41 ± 111.57^Bc^	*p* = 0.826	*p* < 0.0001	*p =* 0.995
1OSP	2644.35 ± 84.42^Ab^	2581.68 ± 109.78^ABb^	2409.78 ± 64.39^Bb^	2086.10 ± 27.84^Cb^	*r* = 0.028	*r* = 0.635**	*r* = n/a
2OSP	2812.73 ± 6.36^Aab^	2694.28 ± 127.17^ABab^	2468.55 ± 50.19^BCb^	2305.01 ± 131.66^Cb^			
1PSP	2693.17 ± 16.34^Ab^	2610.73 ± 44.08^ABb^	2444.35 ± 53.99^Bb^	2238.53 ± 101.98^Cb^			
2PSP	2972.97 ± 181.38^Aa^	2902.23 ± 132.94^Aa^	2739.54 ± 57.27^Aa^	2690.00 ± 126.65^Aa^			
*Cohesiveness*							
Control	−53.79 ± 2.61^Ca^	−50.96 ± 0.84^Ca^	−42.80 ± 2.02^Ba^	−31.47 ± 2.02^Aa^	*p* = 0.978	*p* < 0.0001	*p =* 1.000
1OSP	−67.10 ± 2.87^Bb^	−64.48 ± 1.57^ABb^	−58.67 ± 2.34^Ab^	−57.50 ± 3.02^Ab^	*r* = 0.003	*r* = −0.741**	*r* = n/a
2OSP	−88.28 ± 3.19^Bc^	−86.16 ± 1.19^Bc^	−76.52 ± 4.59^Ac^	−73.75 ± 3.38^Ac^			
1PSP	−71.72 ± 1.93^Bb^	−64.79 ± 5.11^ABb^	−61.49 ± 1.26^Ab^	−59.41 ± 1.30^Ab^			
2PSP	−97.48 ± 1.63^Bd^	−93.75 ± 1.85^Bd^	−84.21 ± 5.37^Ac^	−81.70 ± 2.21^Ac^			
*Index of viscosity (gs)*							
Control	−162.43 ± 8.83^Ba^	−147.35 ± 10.26^Ba^	−91.30 ± 5.69^Aa^	−77.78 ± 4.88^Aa^	*p* = 0.982	*p* < 0.0001	*p =* 1.000
1OSP	−277.68 ± 4.73^Cb^	−228.50 ± 5.60^Bb^	−200.28 ± 2.58^Ab^	−189.79 ± 4.78^Ab^	*r* = 0.002	*r* = −0.767**	*r* = n/a
2OSP	−321.09 ± 13.39^Cc^	−309.11 ± 7.87^BCd^	−281.51 ± 10.07^ABd^	−257.63 ± 9.46^Ad^			
1PSP	−293.33 ± 8.13^Cb^	−260.50 ± 8.35^Bc^	−239.91 ± 6.74^Ac^	−220.71 ± 3.15^Ac^			
2PSP	−344.53 ± 7.92^Cc^	−320.86 ± 13.27^BCd^	−297.34 ± 8.90^Abd^	−290.54 ± 3.67^Ae^			

*Note:* A–D (→): Values with the different capital letters in the same line for each analysis differ significantly (*p* < 0.05), a–e (↓): Values with the different lowercase letters in the same column for each analysis differ significantly (*p* < 0.05). ±Standard deviation.

Abbreviation: S, Samples, ST, Storage time.

** Correlation is significant at the 0.01 level (2‐tailed).

**TABLE 7:** *S. themophilus* and *L. delbrueckii* subsp. *bulgaricus* count of samples during the storage (log cfu/g).

Sample	Storage time (d)				Variation		
	1	4	7	10	S	ST	S × ST
*Count of S. thermophilus*							
Control	8.36 ± 0.03^Aa^	8.14 ± 0.06^Ae^	8.04 ± 0.05^Ab^	7.83 ± 0.55^Ab^	*p* < 0.0001	*p* = 0.710	*p* = 0.352
1OSP	8.55 ± 0.01^Aa^	8.39 ± 0.01^Bd^	8.33 ± 0.01^Cab^	8.33 ± 0.01^Cab^	*r* = 0.599**	*r* = −0.127	*r* = n/a
2OSP	8.64 ± 0.02^Aa^	8.66 ± 0.01^Ac^	8.66 ± 0.01^Aab^	8.66 ± 0.01^Aa^			
1PSP	8.56 ± 0.02^Aa^	8.50 ± 0.01^Bb^	8.48 ± 0.01^Bab^	8.48 ± 0.01^Ba^			
2PSP	8.53 ± 0.66^Aa^	8.82 ± 0.01^Aa^	8.84 ± 0.01^Aa^	8.84 ± 0.01^Aa^			
*Count of L. delbrueckii subsp. bulgaricus*							
Control	7.99 ± 0.01^Aa^	8.00 ± 0.03^Aab^	8.01 ± 0.04^Ab^	7.99 ± 0.01^Acd^	*p* = 0.171	*p* < 0.05	*p* < 0.0001
1OSP	7.89 ± 0.02^Ca^	7.94 ± 0.02^BCbc^	8.01 ± 0.02^ABb^	8.04 ± 0.01^Abc^	*r* = −0.079	*r* = 0.154	*r* = n/a
2OSP	7.95 ± 0.05^Ca^	8.04 ± 0.02^Ba^	8.07 ± 0.02^ABab^	8.12 ± 0.01^Aa^			
1PSP	7.88 ± 0.07^Aa^	7.90 ± 0.02^Ac^	7.92 ± 0.03^Ac^	7.96 ± 0.01^Ad^			
2PSP	7.94 ± 0.05^Ba^	8.00 ± 0.01^Bab^	8.09 ± 0.01^Aa^	8.10 ± 0.02^Aab^			

*Note:* A–C (→): Values with the different capital letters in the same line for each analysis differ significantly (*p* < 0.05), a–d (↓): Values with the different lowercase letters in the same column for each analysis differ significantly (*p* < 0.05). ± Standard deviation.

Abbreviations: S, Samples; ST, Storage time.

** Correlation is significant at the 0.01 level (2‐tailed).

Over the course of the storage of the samples, bacterial counts of *S. thermophilus* and *L. delbrueckii* subsp. *bulgaricus* increased significantly (*p* < 0.05), except in control samples. On the final day of storage, the counts of both bacterial strains were found to be above 8 log CFU/g, and the presence of added lyophilized sweet potatoes (especially orange) was found to have a prebiotic effect on the development of yogurt bacteria.

Omar et al. (2019) reported that the numbers of *L. delbruecii* spp. bulgaricus and *S. thermophilus* in yogurts with sweet potato added increased up to 3 days of storage and then gradually decreased in all applications until the end of storage. They stated that the numbers of *L. delbruecii* spp. *bulgaricus* were 7.80–8.35 log cfu/g on the last day of storage, and the numbers of *S. thermophilus* were between 7.88 and 8.99. These results are parallel to our research.

It was determined that storage time, sample type, and storage time × sample type interactions had significant effects (*p* < 0.0001) on the organic acid values of the samples (Table 8). Storage time interactions also had a highly significant positive correlative effect (*p* < 0.01) on organic acid values. Sample type interactions were found to have a significant positive correlative effect (*p* < 0.05) on tartaric and ascorbic acid values (Table 8).

**TABLE 8:** Organic acid content of samples during the storage period (mg/kg).

Sample	Storage time (d)				Variation		
	1	4	7	10	S	ST	S × ST
*Oxalic acid*							
Control	0.657 ± 0.03^Dc^	1.040 ± 0.07^Cc^	1.893 ± 0.12^Bc^	2.829 ± 0.09^Ac^	*p* < 0.0001	*p* < 0.0001	*p* < 0.0001
1OSP	0.673 ± 0.02^Cc^	0.725 ± 0.03^Cc^	0.923 ± 0.04^Bd^	1.059 ± 0.02^Ad^	*r* = 0.174	*r* = 0.569**	*r* = n/a
2OSP	0.811 ± 0.03^Db^	2.461 ± 0.31^Ca^	4.698 ± 0.23^Ba^	6.021 ± 0.04^Aa^			
1PSP	0.793 ± 0.01^Cb^	0.927 ± 0.02^BCc^	1.050 ± 0.05^Bd^	1.189 ± 0.07^Ad^			
2PSP	1.167 ± 0.05^Da^	1.740 ± 0.09^Cb^	2.852 ± 0.13^Bb^	3.992 ± 0.13^Ab^			
*Tartaric acid*							
Control	1.411 ± 0.03^Ac^	1.423 ± 0.03^Ad^	1.450 ± 0.02^Ad^	1.472 ± 0.02^Ad^	*p* < 0.0001	*p* < 0.0001	*p* < 0.0001
1OSP	1.421 ± 0.01^Dc^	1.830 ± 0.03^Cc^	1.996 ± 0.01^Bc^	2.341 ± 0.05^Ac^	*r* = 0.314*	*r* = 0.483**	*r* = n/a
2OSP	1.501 ± 0.03^Da^	5.881 ± 0.13^Ca^	14.860 ± 0.32^Ba^	26.068 ± 0.08^Aa^			
1PSP	1.431 ± 0.01^Dbc^	1.953 ± 0.02^Cc^	2.373 ± 0.04^Bc^	2.821 ± 0.03^Ac^			
2PSP	1.467 ± 0.02^Dab^	4.811 ± 0.25^Cb^	11.842 ± 0.28^Bb^	16.370 ± 0.43^Ab^			
*Formic acid*							
Control	3079.54 ± 50.62^Ba^	3114.27 ± 14.34^Bb^	3194.31 ± 19.78^ABc^	3264.36 ± 62.14^Ad^	*p* < 0.0001	*p* < 0.0001	*p* < 0.0001
1OSP	2013.44 ± 27.01^Dd^	2706.77 ± 37.42^Cc^	3147.23 ± 63.49^Bc^	3928.76 ± 105.28^Ac^	*r* = 0.133	*r* = 0.556**	*r* = n/a
2OSP	2902.59 ± 3.86^Db^	4838.24 ± 210.71^Ca^	6378.83 ± 61.63^Ba^	10,860.34 ± 77.78^Aa^			
1PSP	1797.89 ± 63.01^De^	2568.33 ± 48.23^Cc^	2873.85 ± 60.10^Bd^	3323.73 ± 17.53^Ad^			
2PSP	2799.38 ± 16.91^Dc^	3160.32 ± 72.14^Cb^	4200.21 ± 33.91^Bb^	6945.33 ± 170.83^Ab^			
*Malic acid*							
Control	34.93 ± 0.25e	46.45 ± 0.41e	73.55 ± 0.41d	96.32 ± 0.35e	*p* < 0.0001	*p* < 0.0001	*p* < 0.0001
1OSP	48.15 ± 0.25c	50.34 ± 0.62d	62.73 ± 0.59e	117.74 ± 0.28c	*r* = 0.174	*r* = 0.569**	*r* = n/a
2OSP	83.73 ± 0.27a	62.45 ± 0.49b	103.42 ± 0.28b	196.11 ± 0.71a			
1PSP	36.83 ± 0.27d	53.53 ± 0.49c	88.42 ± 0.44c	105.27 ± 0.36d			
2PSP	54.93 ± 0.24b	66.42 ± 0.54a	121.44 ± 0.39a	164.45 ± 0.40b			
*Ascorbic acid*							
Control	2.10 ± 0.08^Ab^	2.19 ± 0.01^Ad^	2.31 ± 0.11^Ad^	2.34 ± 0.08^Ae^	*p* < 0.0001	*p* < 0.0001	*p* < 0.0001
1OSP	2.67 ± 0.24^Cb^	2.99 ± 0.10^Cc^	4.74 ± 0.17^Bc^	7.76 ± 0.16^Ac^	*r* = 0.387*	*r* = 0.639**	*r* = n/a
2OSP	4.52 ± 0.59^Da^	6.22 ± 0.16^Ca^	7.60 ± 0.52^Ba^	10.75 ± 0.08^Aa^			
1PSP	2.50 ± 0.30^Cb^	2.66 ± 0.46^Ccd^	4.32 ± 0.42^Bc^	7.16 ± 0.20^Ad^			
2PSP	2.95 ± 0.07^Db^	5.40 ± 0.31^Cb^	6.38 ± 0.36^Bb^	8.73 ± 0.19^Ab^			
*Lactic acid*							
Control	761.73 ± 13.79^Dc^	812.92 ± 14.84^Cd^	948.11 ± 21.69^Bd^	1807.69 ± 8.86^Ae^	*p* < 0.0001	*p* < 0.0001	*p* < 0.0001
1OSP	820.18 ± 20.42^Dc^	934.04 ± 31.51^Cc^	1498.10 ± 14.33^Bb^	2161.88 ± 64.40^Ac^	*r* = 0.165	*r* = 0.837**	*r* = n/a
2OSP	1162.64 ± 35.82^Da^	1449.33 ± 50.78^Ca^	1945.39 ± 60.73^Ba^	2762.83 ± 69.97^Aa^			
1PSP	810.62 ± 25.29^Dc^	903.98 ± 24.66^Ccd^	1327.08 ± 38.65^Bc^	1995.77 ± 16.46^Ad^			
2PSP	984.83 ± 10.59^Db^	1158.94 ± 69.71^Cb^	1393.15 ± 85.11^Bbc^	2316.30 ± 28.29^Ab^			
*Sitric acid*							
Control	21.44 ± 1.86^Cc^	35.82 ± 3.40^Bd^	38.59 ± 4.64^Be^	82.63 ± 3.14^Ad^	*p* < 0.0001	*p* < 0.0001	*p* < 0.0001
1OSP	43.47 ± 4.47^Db^	59.34 ± 2.82^Cc^	79.57 ± 2.34^Bc^	117.59 ± 3.99^Ac^	*r* = 0.328*	*r* = 0.597**	*r* = n/a
2OSP	93.32 ± 2.99^Da^	109.88 ± 3.59^Ca^	158.66 ± 4.73^Ba^	292.07 ± 5.98^Aa^			
1PSP	36.37 ± 4.03^Cb^	43.73 ± 6.50^Cd^	67.99 ± 1.90^Bd^	110.90 ± 5.16^Ac^			
2PSP	85.31 ± 2.63^Da^	95.83 ± 3.08^Cb^	116.05 ± 1.82^Bb^	189.15 ± 6.07^Ab^			
*Sucsinic acid*							
Control	1049.56 ± 9.10^Dd^	1341.16 ± 53.52^Cd^	2677.47 ± 33.69^Be^	4402.96 ± 9.68^Ae^	*p* < 0.0001	*p* < 0.0001	*p* < 0.0001
1OSP	1279.33 ± 15.26^Dc^	1607.27 ± 19.85^Cc^	4771.72 ± 68.45^Bc^	7750.21 ± 45.12^Ac^	*r* = 0.274	*r* = 0.734**	*r* = n/a
2OSP	3268.68 ± 167.50^Da^	5773.23 ± 199.40^Ca^	8787.63 ± 31.81^Ba^	14,808.33 ± 257.11^Aa^			
1PSP	1173.51 ± 49.21^Dcd^	1370.63 ± 40.29^Ccd^	3906.87 ± 48.71^Bd^	6499.82 ± 5.07^Ad^			
2PSP	2490.23 ± 23.89^Db^	3798.21 ± 15.42^Cb^	6094.73 ± 10.60^Bb^	12,889.45 ± 172.36^Ab^			
*Fumaric acid*							
Control	1.14 ± 0.02^Bc^	1.21 ± 0.02^Bd^	1.33 ± 0.04^Ad^	1.34 ± 0.04^Ae^	*p* < 0.0001	*p* < 0.0001	*p* < 0.0001
1OSP	1.29 ± 0.02^Cb^	1.51 ± 0.04^Bb^	1.55 ± 0.04^Bc^	1.73 ± 0.03^Ac^	*r* = 0.408**	*r* = 0.566**	*r* = n/a
2OSP	1.55 ± 0.05^Da^	1.69 ± 0.03^Ca^	2.11 ± 0.03^Ba^	2.27 ± 0.02^Aa^			
1PSP	1.34 ± 0.05^Cb^	1.41 ± 0.03^Cc^	1.51 ± 0.04^Bc^	1.64 ± 0.01^Ad^			
2PSP	1.49 ± 0.05^Ca^	1.58 ± 0.02^BCb^	1.73 ± 0.13^Bb^	1.97 ± 0.02^Ab^			

*Note:* A–D (→): Values with the differ capital letters in the same line for each analysis differ significantly (*p* < 0.05), a–e (↓): Values with the differ lowercase letters in the same column for each analysis differ significantly (*p* < 0.05). ± Standard deviation.

Abbreviations: S, Samples, ST, Storage time.

* Correlation is significant at the 0.05 level (2‐tailed).

** Correlation is significant at the 0.01 level (2‐tailed).

The addition of sweet potatoes had a favorable effect on the samples' organic acid values, and these values increased as the added concentration increased (*p* < 0.05). Products made with a 2% orange potato addition showed the largest rise in organic acid values during storage, followed by samples made with a 2% purple sweet potato and a 1% orange sweet potato addition (Table 8).

Furthermore, the organic acid values of all samples increased during the storage period (*p* < 0.05), and the samples with the highest increase in organic acid values during storage were found to be those produced with the addition of 2% orange potatoes. Compared to control samples, increases in the organic acid values during the storage of samples with the addition of orange and purple sweet potatoes were observed for tartaric, ascorbic, citric, succinic, oxalic, formic, malic, and lactic acid, in order of largest to smallest increases.

Lyophilized sweet potato had a prebiotic effect on the growth of yogurt bacteria due to both its high fiber content and rich nutrients. Therefore, the addition of sweet potato caused yogurt bacteria to display more activity and to survive in higher numbers for longer periods of time, resulting in higher organic acid production during the storage period.

Organic acids have positive effects on the taste and aroma of fermented foods such as yogurt as well as on the shelf life of the products. The variety of organic acids offers a richer flavor, improves the taste and aroma of products, and positively affects consumer preferences.

The addition of orange and purple sweet potatoes increased the mineral content of the yogurt samples. In particular, the addition of purple sweet potatoes was found to have a greater effect on the increase in mineral content compared to orange sweet potatoes. Among the samples, the lowest mineral content was observed in the control samples, while the highest mineral content was found in samples with the addition of 2% purple sweet potatoes. Added sweet potatoes were found to enrich yogurt particularly in terms of Se, Fe, and K (Table 9).

**TABLE 9:** Mineral matter analysis results of yogurt samples (ppb).

	B	Na	Mg	K	Ca
Control	523.61	205,492.27	130,536.16	2,068,741.43	112,564.88
1OSP	425.74	201,258.18	166,324.25	2,154,801.25	114,698.44
2OSP	603.57	225,471.02	180,154.25	2,365,874.25	120,367.74
1PSP	704.58	230,578.24	170,547.11	2,547,998.15	121,875.11
2PSP	801.47	250,078.36	203,691.47	2,765,987.42	125,981.47
	**Mn**	**Fe**	**Zn**	**Se**	
Control	65.14	662.54	3642.47	10.36	
1OSP	308.25	865.48	3766.24	12.69	
2OSP	466.47	1095.87	3804.19	13.50	
1PSP	796.18	1133.75	3849.55	13.13	
2PSP	833.65	1549.86	3909.08	14.29	

## 4 Conclusions

When lyophilized sweet potatoes were added to the samples, the organic acid values rose. Organic acid values increased parallel to the rate of the added sweet potatoes. The addition of orange potatoes was more effective on organic acid values than the addition of purple potatoes. Furthermore, the addition of sweet potatoes enriched the yogurt samples in terms of mineral content.

At the end of storage, the highest titratable acidity (1.56%) and the lowest pH value (4.26), the highest firmness (229.07 g) and consistency (2690.00 g) and the cohesiveness (−81.70) and viscosity index (−290.54 g) values were determined in samples produced with a 2% orange sweet potato addition. On the other hand, the highest dry matter (8.45%), water holding capacity (50.90), DPPH radical scavenging effect (112.97%), and total phenolic substance values (2.64 mg GAE/g) were found in samples produced with a 2% purple sweet potato addition.

During storage, the count of *L. delbrueckii* subsp. bulgaricus (0.174 log cfu/g) increased the most in samples with 2% orange sweet potato added, while the number of *S. thermophilus* (0.30 log cfu/g) increased in samples with 2% purple sweet potato added. In addition, the highest oxalic (6.021 mg/kg), tartaric (26.068 mg/kg), formic (10,860 mg/kg), malic (196.11 mg/kg), ascorbic (10.75 mg/kg), and lactic acid (2762.83 mg/kg) amounts were determined in yogurts with 2% orange sweet potato added. On the other hand, it was determined that the samples richest in terms of mineral content were yogurts produced with the addition of 2% purple sweet potato, and the highest minerals were determined as potassium (2,765,987.42 ppb), sodium (250,078 ppb), and magnesium (203,691.47 ppb), respectively.

When the quality criteria of yogurt were evaluated in terms of textural and microbiological properties and organic acids that form its taste and aroma, it was determined that the addition of orange sweet potato gained importance, and when it was evaluated in terms of % DPPH radical scavenging effect, total phenolic substance, and mineral substance content, the addition of purple sweet potato gained importance.

Functional meals are becoming more and more in demand from customers today. Orange and purple sweet potatoes were added to yogurt samples to improve their content of organic acids, fiber, vitamins, and minerals. They also provided a significant amount of phenolic compounds. Additionally, they offered a great deal of promise in terms of antioxidant activity. Yogurts with added sweet potatoes are seen to offer significant promise in terms of consumer preferences due to their enhanced functional and nutritional qualities. The addition of sweet potato has shown a prebiotic effect that supports the development of probiotic bacteria. As a result, it will also have an effect on postbiotic metabolites synthesized by probiotics, whose value for human health has begun to be revealed in recent years.

This study has shown that adding both types of sweet potatoes provides significant functionality to yogurt and increases its nutritional value. Future studies should concentrate on the impact of sweet potato addition on probiotic bacteria development and postbiotic metabolite production.

## Author Contributions

**Mehmet KIlinç:** conceptualization (equal), funding acquisition (equal), project administration (equal). **Ayşe Janseli Denizkara:** conceptualization (equal), formal analysis (equal), methodology (equal), resources (equal), writing – review and editing (equal). **Gökhan Akarca:** conceptualization (equal), data curation (equal), methodology (equal), resources (equal), software (equal), validation (equal), writing – original draft (equal).

## Ethics Statement

The authors have nothing to report.

## Consent

The authors have nothing to report. Consent to Participate: The corresponding author and all the co‐authors participated in the preparation of this manuscript.

## Conflicts of Interest

The authors declare no conflicts of interest.

## Data Availability

The original data with the respective analysis corresponding to the results shown in this work are available up to reasonable requirements.
